# Genetic polymorphism of merozoite surface protein-1 and merozoite surface protein-2 in *Plasmodium falciparum *field isolates from Myanmar

**DOI:** 10.1186/1475-2875-9-131

**Published:** 2010-05-17

**Authors:** Jung-Mi Kang, Sung-Ung Moon, Jung-Yeon Kim, Shin-Hyeong Cho, Khin Lin, Woon-Mok Sohn, Tong-Soo Kim, Byoung-Kuk Na

**Affiliations:** 1 Department of Parasitology, Brain Korea 21 Biomedical Center, and Institute of Health Sciences, Gyeongsang National University School of Medicine, Jinju 660-751, South Korea; 2 Division of Malaria and Parasitic Diseases, National Institute of Health, Korea Centers for Disease Control and Prevention, Seoul 122-701, South Korea; 3 Vector Borne Diseases Control Project, Department of Health, 36 Theinbyu Road, Mandalay, Myanmar; 4 Department of Parasitology and Inha Research Institute for Medical Sciences, Inha University School of Medicine, Incheon 400-712, South Korea

## Abstract

**Background:**

Merozoite surface protein-1 (MSP-1) and MSP-2 of *Plasmodium falciparum *are potential vaccine candidate antigens for malaria vaccine development. However, extensive genetic polymorphism of the antigens in field isolates of *P. falciparum *represents a major obstacle for the development of an effective vaccine. In this study, genetic polymorphism of MSP-1 and MSP-2 among *P. falciparum *field isolates from Myanmar was analysed.

**Methods:**

A total of 63 *P. falciparum *infected blood samples, which were collected from patients attending a regional hospital in Mandalay Division, Myanmar, were used in this study. The regions flanking the highly polymorphic characters, block 2 for MSP-1 and block 3 for MSP-2, were genotyped by allele-specific nested-PCR to analyse the population diversity of the parasite. Sequence analysis of the polymorphic regions of MSP-1 and MSP-2 was also conducted to identify allelic diversity in the parasite population.

**Results:**

Diverse allelic polymorphism of MSP-1 and MSP-2 was identified in *P. falciparum *isolates from Myanmar and most of the infections were determined to be mixed infections. Sequence analysis of MSP-1 block 2 revealed that 14 different alleles for MSP-1 (5 for K1 type and 9 for MAD20 type) were identified. For MSP-2 block 3, a total of 22 alleles (7 for FC27 type and 15 for 3D7 type) were identified.

**Conclusion:**

Extensive genetic polymorphism with diverse allele types was identified in MSP-1 and MSP-2 in *P. falciparum *field isolates from Myanmar. A high level of mixed infections was also observed, as was a high degree of multiplicity of infection.

## Background

Malaria is a major human health-threatening disease, which resulting in approximately 200-300 million clinical cases and 1-3 million deaths each year worldwide. *Plasmodium falciparum *causes the most severe form of the disease and is responsible for most malaria morbidity and almost all malaria mortality. Despite enormous efforts for malaria control and prevention, multiple factors, including insecticide resistance in the mosquito vectors, the lack of effective vaccines, and the emergence and rapid spread of drug-resistant strains, are contributing to the global worsening of the malaria situation. Therefore, there is an urgent need for the development of effective malaria vaccine. However, extensive genetic diversity in natural parasite populations is a major obstacle for the development of an effective vaccine against the human malaria parasite, since antigenic diversity limits the efficacy of acquired protective immunity to malaria [1-3]. Therefore, it is important to investigate the genetic diversity of malaria parasites, particularly the genetic diversity of vaccine candidate antigens, in different geographic regions to develop effective malaria vaccines.

Merozoite surface protein-1 (MSP-1) of *P. falciparum *is a major surface protein, with an approximate molecular size of 190 kDa, that plays an important role in erythrocyte invasion by the merozoite [4]. The protein is a principal target of human immune responses [5-7] and is a promising candidate for a blood stage subunit vaccine [4,8]. The MSP-1 gene has 7 variable blocks that are separated either by conserved or semi-conserved regions. Block 2, a region near the N-terminal of the MSP-1 gene, is the most polymorphic part of the antigen and appears to be under the strongest diversifying selection within natural populations [4]. Up to now, four different allelic types of block 2 have been identified: MAD20, K1, RO33, and MR [9-12].

MSP-2 of *P. falciparum *is another leading candidate antigen for subunit malaria vaccine [13]. It comprises highly polymorphic central repeats flanked by unique variable domains and conserved N- and C-terminal domains [1,14]. The MSP-2 alleles generally fall into two allelic types, FC27 and 3D7, which differ considerably in the dimorphic structure of the variable central region, block 3. Due to their polymorphic features, the MSP-1 and MSP-2 genes have been employed as polymorphic markers in studies of malaria transmission dynamics in natural isolates of *P*. *falciparum*.

The morbidity and mortality rates due to malaria have been declining gradually in recent years in Myanmar, but malaria still remains one of the most serious problems threatening human health in the country, resulting in approximately 60% of malaria deaths in the South-East Asia region [15]. Information on the nature and extent of population diversity within malaria parasites circulating in the country is essential not only for understanding the mechanism underlying the pathology of malaria but also for establishing a proper control strategy. However, only limited data are available on the genetic diversity of *P. falciparum *populations of the country. This study was designed to analyse the genetic diversity of MSP-1 and MSP-2 in field isolates of *P. falciparum *collected in a rural area outside of Mandalay, Myanmar.

## Methods

### Blood samples and genomic DNA extraction

A total of 63 *P. falciparum *infected blood samples used in this study were collected from patients attending the Wet-Won station hospital, Pyin Oo Lwin Township, Mandalay Division, Myanmar during 2004-2006 [16]. All blood samples were collected after informed consent and the use of the samples for this study was approved by the Department of Health, The Union of Myanmar, and the Ethic Committee of the Centers for Disease Control and Prevention, Korea. Genomic DNA was extracted from 100 μl of whole blood sample by using a QIAamp Blood Kit (Qiagen, Valencia, CA, USA) following the manufacturer's instruction.

### Allelic typing of MSP-1 and MSP-2

The oligonucleotide primers specific for the polymorphic regions (block 2 of MSP-1 and block 3 of MSP-2) were designed as described previously [17]. The two genes were amplified by nested PCR. An initial amplification of the outer regions of the two genes was followed by a nested PCR with family-specific primer pairs. All reactions were carried out in a final volume of 40 μl containing 0.2 mM dNTP, 1 μM of each primer, and 2.5 U of Ex *Taq *DNA polymerase (Takara, Otsu, Japan). In the first round reaction, 4 μl of genomic DNA was added as a template. In the nested reaction, 1 μl of the first PCR product was added. Each amplification profile consisted of initial denaturation at 94°C for 5 min, followed immediately by 30 cycles of 94°C for 1 min, 55°C for 1 min, and 72°C for 2 min. The final cycle had a prolonged extension at 72°C for 10 min. Each PCR product was electrophoresed on 1.5% agarose gels and the DNA was visualized by ultraviolet transillumination after staining with ethidium bromide. The number and size of the resulting amplified products were analysed.

### Allelic distribution and multiplicity of infection

The prevalence of each allelic type was determined as the presence of PCR products for the type in the total number of amplified bands for the corresponding locus. The multiplicity of infection (MOI), or complexity of infection, was estimated by the average number of PCR fragments per infected individual, as described previously [18,19].

### Sequencing analysis of MSP-1 and MSP-2

For sequence analysis of MSP-1 and MSP-2, all PCR products (128 for MSP-1 and 148 for MSP-2) obtained by allelic typing PCR were purified from the gel and cloned into the pGEM-T Easy vector (Promega, Madison, WI, USA). Each ligation mixture was transformed into *E. coli *DH5α competent cells and positive clones were screened for the presence of plasmid with the appropriate insert. Sequencing reactions were performed using the BigDye Terminator Cycle Sequencing Ready Reaction Kit in an ABI 377 automatic DNA sequencer (Applied Biosystems, Foster City, CA, USA). To verify the sequences, sequence analysis was performed by analysing at least two plasmid clones containing each gene insert. Analysis of the primary structures of the deduced amino acid sequences was done with DNASTAR (DNASTAR, Madison, WI, USA). Nucleotide sequences reported in this paper are available in the GenBank database under accession numbers EU445555-EU445557, EU445559-EU445566, GQ861442-GQ861443, and GQ861445 for MSP-1, and numbers EU647447-EU647468 for MSP-2.

## Results

### Allelic polymorphism of MSP-1 and MSP-2

Allele typing analysis displayed the highly polymorphic nature of *P. falciparum *Myanmar isolates with respect to MSP-1 and MSP-2. In MSP-1, K1 and MAD20 allele types were identified, but neither RO33 nor MR allele type were identified. K1 allele type was identified in 46 blood samples but the majority (63.5%, 40/63) occurred as mixed infections with the MAD20 allele type. The remaining 17 samples were identified as single infection by the MAD20 allele type (Table 1). The length variants of the amplified product were approximately 120-210 bp for K1 and 140-250 for MAD20. For MSP-2, both FC27 and 3D7 allele types were identified among the isolates. The frequency of samples having only FC27 allele type was 12.7% (8/63), while the frequency having only 3D7 allele type was 19.0% (12/63). Both alleles were found in 68.3% (43/63) of the samples (Table 1). The length variants of the amplified product were about 260-500 bp for FC27 and 400-610 bp for 3D7. A large proportion of isolates (50/63 for MSP-1 and 55/63 for MSP-2) showed more than 2 PCR products for each locus, as visualized on agarose gel as a double band or multiple bands. The MOI was 2.03 and 2.35 for MSP-1 and MSP-2, respectively. These results collectively suggest that diverse allelic polymorphism of MSP-1 and MSP-2 was identified in *P. falciparum *isolates from Myanmar and that most of the infections were mixed.

**Table 1:** Allele typing and diversity profiles of *P. falciparum *isolates from Myanmar based on genetic diversity of MSP-1 and MSP-2.

	No. of samples	PCR product size (bp)	Frequency (%)	Multiplicity of infection (MOI)
MSP-1				
K1	6	120-210	9.5	1.50
MAD20	17	140-250	27.0	1.41
K1 + MAD20	40		63.5	2.38
Total	63		100	2.03
				
MSP-2				
FC27	8	260-500	12.7	1.63
3D7	12	400-610	19.0	1.58
FC27 + 3D7	43		68.3	2.70
Total	63		100	2.35

### Sequence analysis of MSP-1

**Figure 1:**
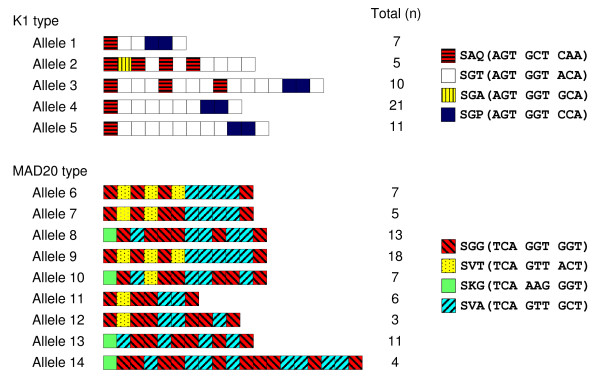
**Schematic representation of MSP-1 block 2 of *P. falciparum *isolates from Myanmar**. A total of 15 different alleles, including 5 for K1 type and 9 for MAD20 type, were identified by sequence analysis of MSP-1 block 2. K1 type alleles differ in the number and arrangement of SAQ, SGT, SGA and SGP motifs, while MAD20 type alleles differ in the number and arrangement of SGG, SVT, SKG, and SVA motifs. The total number of each allele is indicated.

A total of 14 different alleles of MSP-1 were recognized by sequence analysis of the MSP-1 block 2 region (Figure 1). Sequence analysis also confirmed that they were grouped into K1 and MAD20. The MAD20 allele type was more diverse with nine different alleles, compared to five from the K1 allele type. A limited number of different tripeptide repeat units (4 for K1 and 4 for MAD-20) were identified in Myanmar isolates. Most sequence variation in block 2 of MSP-1 is actually created by rearrangements of a limited number of building blocks. The overall structure of some repeat arrays (for example, alleles 4 and 5; alleles 6, 7, and 9) is remarkably conserved. In the K1 type alleles, the tripeptide repeat region always started with SAQ and terminated with SGT, regardless of differences in the number of tripeptide repeats. Most diversity was due to duplications or deletions of the repeat motifs SAQ, SGT and SGP. In the MAD20 type alleles, the repeat region started with one of two different tripeptide sequences, SGG or SKG, but always ended with the identical hexapeptide sequence, SVASGG. The diversity of the MAD20 allele type was also caused primarily by differences in repetitions of SGG, SVT and SVA. The overall frequencies of individual alleles in *P. falciparum *Myanmar isolates were broadly distributed throughout the allele types, but allele 4 (38.9%) for K1 type and allele 9 (24.3%) for MAD20 type were predominant.

### Sequence analysis of MSP-2

**Figure 2:**
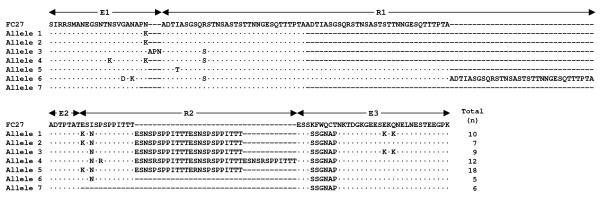
**Sequence alignment of the predicted amino acid sequences of FC27 allelic types MSP-2 from *P. falciaprum *isolates from Myanmar**. The central variable region of allelic types is compared to the reference FC27 (GenBank accession number J03828). Identical residues are indicated by dots. Dashes represent gaps introduced to maximize the alignment. The family-specific region (E1-E3) and the two tandem repeats (R1 and R2) are indicated. The total number of each allele is indicated.

**Figure 3:**
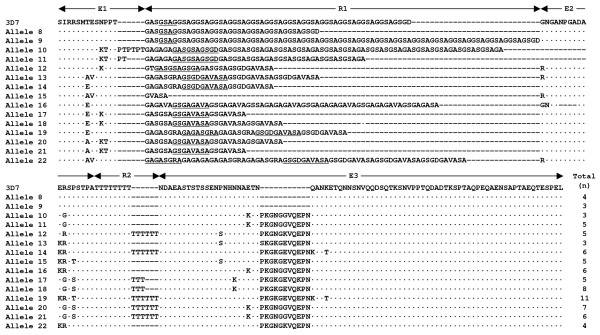
**Sequence alignment of the predicted amino acid sequences of 3D7 allelic types MSP-2 from *P. falciaprum *isolates from Myanmar**. The central variable region of allelic types is compared to the reference 3D7 (GenBank accession number X53832). Identical residues are indicated by dots. Dashes represent gaps introduced to maximize the alignment. The family-specific region (E1-E3) and the two tandem repeats (R1 and R2) are indicated. Each repeat unit in R1 is underlined. The poly-threonine stretch is highlightened by shading. The total number of each allele is indicated.

Sequence analysis of MSP-2 block 3 revealed that a total of 22 distinguishable allele sequences were identified among *P. falciparum *isolates from Myanmar. Both types of FC27 and 3D7, which differ considerably in the dimorphic structure of the variable central region, were identified. A total of seven alleles for FC27 and 15 alleles for 3D7 were observed. The FC27 family showed varying numbers of structurally conserved R1 (96 bp) and R2 (36 bp) repetitive regions. All FC27 alleles had repetitive sequences that were related, but not identical to each other (Figure 2). The R1 regions of all compared alleles of FC27 type consist of 1-3 copies of the family-specific repeat unit, ADTIASGSQSSTNSASTSTTNNGESQTTTPTA or its variants with the same number of amino acid residues. The 21 bp E2 repeat unit was well conserved in all alleles. The R2 region showed polymorphic nature with different number of copies (0-4 copies) of 36 bp repeat unit. All FC27 type alleles shared a non-synonymous substitution of 6 amino acids (SSGNAP) in the E3 region, which is resulted from a single indel event leading to a change in the open reading frame [14]. The PNA motif at the 3' end of E1 was duplicated in allele 3. Several nucleotide substitutions, all leading to non-synonymous amino acid changes in E1, R1, R2, and E3 regions, also contributed to the allelic diversity of FC27 alleles. The 3D7 allele type showed more complicated variations than FC27 allele type. The R1 had an extremely polymorphic character, consisting of multiple copies of 4-10 amino acids that differed in number, sequence, and length (Figure 3). The R2 poly-threonine stretch, which is typical for all alleles of the 3D7 family, also showed polymorphic patterns among the alleles with different numbers (8-14) of threonine residues. Several non-synonymous amino acid substitutions were also identified in other family specific regions (E1, E2, and E3) and the variation is much greater than FC27 type alleles. Interestingly, duplication of PT motif at the 3' end of block 2 was identified in alleles 10 and 11. Deletion of 11 amino acids (PKGNGGVQEPN) in E3 region, which is relatively common in field isolates from other geographical regions [14], was also found in alleles 8 and 9.

## Discussion

The genetic structure of *P. falciparum *populations plays a highly important role in the natural acquisition of immunity in malarial infections [2,20]. Therefore, knowledge of the genetic structure of these populations is necessary to develop strategies to control the disease, including the design of effective vaccines against *P. falciparum*. In this study, genetic polymorphism of two merozoite surface proteins, MSP-1 and MSP-2, of 63 *P. falciparum *isolates collected in Myanmar, where malaria is endemic or hypoendemic, was analysed [15]. To our knowledge, no such study has been done in Myanmar to date, and therefore this study provides the first estimate of the genetic diversity of *P. falciparum *wild-type isolates circulating in Myanmar.

Allele-specific PCR typing of MSP-1 (block 2) and MSP-2 (block 3) showed that *P. falciparum *populations in Myanmar have a highly complex genetic diversity. For MSP-1, both types of K1 and MAD20 with different length of amplified products (120-210 bp for K1 and 140-250 bp for MAD20) were identified. Most of the isolates (63.5%) were mixed infections which harbored both allele types. Several similar studies in different geographic areas which used block 2 of MSP-1 as a polymorphic marker reported important variations in the frequency of the genotypes. MAD20 (57/63, 90.5%) was the predominant allele in the *P. falciparum *population in Myanmar, which is consistent with the situations in Thailand, Iran, Pakistan and Colombia [21-24]. On the other hand, in studies in French Guiana, Kenya and Peru, MAD20 is the less frequent type and K1 is the most frequent [11,25,26]. Allele typing for MSP-2 also showed that both FC27 and 3D7 allele types were identified among the isolates. The frequency of FC27 and 3D7 allele type was 80.9% (51/63) and 87.3% (55/63), respectively, but a high proportion of the isolates (68.3%, 43/63) contained both allele types. Similar frequency patterns are observed in Thailand, Iran, Pakistan and Cameroon [21,23,24,27], but not in Brazil, where FC27 type is more prevalent [28]. These results collectively suggest that diverse allelic variations of MSP-1 and MSP-2 exist in *P. falciparum *Myanmar isolates and that most of the infections were mixed. This is similar to observations made in other endemic areas [11,17,21,22,27-29].

To further investigate the allelic diversity of MSP-1 and MSP-2 in *P. falciparum *isolates from Myanmar, sequence analysis of MSP-1 and MSP-2 was performed. Sequence analysis of MSP-1 block 2 showed that a total of 14 alleles of MSP-1, 5 for K1 type and 9 for MAD20 type, were identified. Allelic diversity of MSP-1 block 2 in *P. falciparum *Myanmar isolates was due to different numbers of unique tripeptide repeats, which is similar to previous studies [14,30]. Sequence analysis of MSP-2 block 3 also showed high allelic diversity, with seven alleles for FC27 and 15 alleles for 3D7. As reported in previous studies on parasites from different geographic origins [14,28,30], the sequences belonging to the FC27 family of *P. falciparum *isolates from Myanmar were generally conserved but varied in the number of repeats. The 3D7 displayed more extensive sequence diversity. Besides the major polymorphic characters in the R1 and R2 regions, several non-synonymous amino acid substitutions were identified in family specific regions (E1, E2, and E3) of 3D7 type alleles and the variations make the genetic diversity of 3D7 allele type much greater than FC27 type alleles. Interestingly, duplication of PT motif at the 3' end of block 2 was identified in two 3D7 type alleles (alleles 10 and 11). This proliferation of the PT motif had been identified in non-Asian parasites previously [14], but it is the first description of PT duplication in Asian parasite. MSP-2 was more polymorphic than the MSP-1 in *P. falciparum *isolates from Myanmar, which is also consistent with previous studies [17,21,27,31,32].

Although it seems likely that nonreciprocal recombination events, such as replication slippage and gene conversion, during the mitotic (asexual) replication of the parasite also play a plausible role in creating allele variation [6,33], allelic diversity of *P. falciparum *MSP-1 and MSP-2 is mainly generated by meiotic recombination events involving genetically distinct parasite clones that infect the same mosquito vector [34,35]. Therefore, the proportion of mixed infections and the number of clones per individual is one of the pre-requisites to generate new genotypes and to increase the diversity of the parasitic population [36]. Multiple clonal infections with different genotypes of *P. falciparum *were identified among Myanmar *P. falciparum *isolates in a high proportion (79.4% for MSP-1 and 87.3% for MSP-2). And a high level of MOI (2.03 for MSP-1 and 2.35 for MSP-2) was also found. Sequence analysis of MSP-1 and MSP-2 also showed that diverse alleles (14 for MSP-1 and 22 for MSP-2) were identified among the isolates. Although direct comparison could be impossible due to the different size of blood samples used in each study, this is less than holoendemic areas such as Senegal (33 for MSP-1 and 17 for MSP-2) [37], Uganda [38] and Gabon (25 for MSP-1 and 19 for MSP-2) [17] but more than low endemic Asian countries including Thailand (10 for MSP-1 and 17 for MSP-2) [21] and Iran (9 for MSP-1 and 11 for MSP-2) [39]. These extensive allelic variations were also identified in circumsporozoite protein (CSP), MSP-1, and MSP-3α [16] and apical membrane antigen-1 (AMA-1) [40] of *P. vivax *isolates from Myanmar. The unique geographic location of Myanmar, which is surrounded by five neighbouring malaria endemic countries, appears to contribute to the large diversity of parasite genotypes in this country. Migration of people within the country and between neighbouring countries may also introduce a *P. falciparum *population with different alleles to the country, resulting in an extensive sequence variation in the parasite. Further studies associated with antibody responses against MSP-1 and MSP-2 in Myanmar patients are needed to evaluate the impact of this polymorphism on the immune response to the antigens, since the genetic diversity would not necessarily reflect selection acting at protein level. Studies using a larger number of blood samples collected from different geographic areas in Myanmar are also required not only to determine the nationwide parasite heterogeneity and detailed malaria epidemiology but also to implement malarial control programmes in the country.

## Conclusion

A major finding of this study was that *P. falciparum *field isolates in Myanmar exhibited a high degree of genetic polymorphism in MSP-1 and MSP-2. Moreover, most of the infections were mixed with a high level of MOI. These results collectively suggested the highly complex population structure of the parasite in Myanmar.

## Competing interests

The authors declare that they have no competing interests.

## Authors' contributions

JMK and SUM conducted the molecular genetic studies and analysed the data. JYK, SHC, and KL participated in the blood sampling for this study. WMS and TSK assisted in writing and editing the manuscript. BKN designed the study, supervised the study process and wrote the manuscript. All authors read and approved the final manuscript.
